# Shared and unique characteristics of metabolic syndrome in psychotic disorders: a review

**DOI:** 10.3389/fpsyt.2024.1343427

**Published:** 2024-03-04

**Authors:** Luigi F. Saccaro, Alberto Aimo, Giorgia Panichella, Othman Sentissi

**Affiliations:** ^1^ Psychiatry Department, Faculty of Medicine, University of Geneva, Geneva, Switzerland; ^2^ Psychiatry Department, Geneva University Hospital, Geneva, Switzerland; ^3^ Interdisciplinary Center for Health Sciences, Scuola Superiore Sant’Anna, Pisa, Italy; ^4^ Cardiology Division, Fondazione Toscana Gabriele Monasterio, Pisa, Italy

**Keywords:** psychosis, bipolar disorder, schizophrenia, schizoaffective disorder, psychiatry, cardiology, BMI, weight

## Abstract

**Introduction:**

People with psychosis spectrum disorders (PSD) face an elevated risk of metabolic syndrome (MetS), which may reduce their life expectancy by nearly 20%. Pinpointing the shared and specific characteristics and clinical implications of MetS in PSD is crucial for designing interventions to reduce this risk, but an up-to-date review on MetS across the psychosis spectrum is lacking.

**Methods:**

This narrative review fills this gap by examining the clinical literature on characteristics and implications of MetS in both distinct PSD and transdiagnostically, i.e., across traditional categorical diagnoses, with a focus on psychiatric and cardio-metabolic management.

**Results:**

We discuss common and specific characteristics of MetS in PSD, as well as factors contributing to MetS development in PSD patients, including unhealthy lifestyle factors, genetic predisposition, pro-inflammatory state, drugs consumption, antipsychotic medication, and psychotic symptoms. We highlight the importance of early identification and management of cardio-metabolic risk in PSD patients, as well as the existing gaps in the literature, for instance in the screening for MetS in younger PSD patients. We compare hypotheses-generating clinical associations and characteristics of MetS in different PSD, concluding by reviewing the existing recommendations and challenges in screening, monitoring, and managing MetS in PSD.

**Conclusion:**

Early identification and management of MetS are crucial to mitigate the long-term cardio-metabolic toll in PSD patients. Interventions should focus on healthy lifestyle and appropriate pharmacological and behavioral interventions. Further translational and clinical research is needed to develop targeted interventions and personalized treatment approaches for this vulnerable population, aiming at improving physical health and overall well-being.

## 1 Introduction: a transdiagnostic approach to metabolic syndrome in psychosis

Metabolic syndrome (MetS), also known as syndrome X, is a cluster of interconnected physiological, clinical, and metabolic risk factors associated with an elevated risk for cardiovascular disease, type 2 diabetes mellitus (T2DM), and overall mortality (1). Its defining criteria, often based on the International Diabetes Federation guidelines, encompass components such as obesity, hypertension, dyslipidemia, and insulin resistance (1), although other definitions exist.

Growing evidence has highlighted a bidirectional relationship between MetS and the prevalence and severity of various psychiatric disorders (2), particularly those falling within the psychosis spectrum (1–3). Psychosis-spectrum disorders (PSD) encompass serious and common psychiatric conditions such as schizophrenia, schizoaffective disorder, and bipolar disorder (BD) (3). Individuals affected by these disorders often exhibit an increased vulnerability to cardiometabolic comorbidities, including obesity, insulin resistance, hypertension, diabetes, and ultimately, MetS (4–7).

Not only does MetS contribute to substantial morbidity, elevated costs for public health systems, and premature mortality, thereby underscoring the need for tailored and comprehensive treatment strategies that address both psychiatric and metabolic concerns, but it also reveals a critical imperative for a holistic and integrated approach to healthcare that transcends traditional boundaries between mental and physical health domains.

While existing literature has predominantly focused on MetS in individual psychiatric disorders, there remains a notable gap in scholarship pertaining to a comprehensive evaluation of both shared characteristics and distinctive aspects specifically within PSD.

This gap persists, despite the growing body of evidence that underscores the clinical and research advantages derived from adopting a transdiagnostic and dimensional perspective (8). This innovative framework places a heightened emphasis on investigating features that transcend specific psychiatric diagnoses (9, 10), such as MetS, thereby fostering an integrative perspective that surpasses traditional reductionistic paradigms. This approach is supported by the significant overlap across psychiatric disorders in symptoms (11, 12), genetics (13), and high comorbidity (14), suggesting that traditional categorical classifications may not be the most appropriate for investigating features of psychiatric disorders.

Therefore, the present narrative review examines the existing literature on common and distinct clinical implications and characteristics of MetS across the spectrum of psychotic disorders.

## 2 Shared and distinct epidemiological characteristics of psychosis spectrum disorders

In the general population, MetS prevalence ranges from 10 to 20% in adults and 0-19% in children (15, 16); it is most common in South Asians (17) and African Americans (18), while it is lowest in Europeans (17).

MetS prevalence in PSD has been estimated to be as high as 30% (19), while, as a comparison, it was found to be around 20% in BPD patients (16). A meta-analysis on 1,009 treatment naïve patients with first-episode psychosis (FEP) found a MetS prevalence of 13% (20), while another metanalysis founds MetS in 20% of the 800 untreated schizophrenia patients (19). Males and Asian patients seem to have the highest prevalence of MetS (20).

However, these rates are difficult to compare with the general population, since FEP patients are typically relatively young. Interestingly, a case-control study found a similar MetS prevalence between 303 drug-naïve FEP patients and 153 controls. Despite this, 61% of FEP patients displayed at least one MetS component compared to 37% of controls, along with more prevalent alterations in other cardiovascular risk factors, suggesting an increased risk in this populations (21).

Among specific diagnostic groups of PSD patients, the highest prevalence of MetS seems to be found among patients with BD or schizoaffective disorder (22, 23), with prevalences as high as 67% in BD patients (22, 24, 25), and up to 70% among schizoaffective disorder ones (26).

MetS prevalence in schizophrenia ranges from 10% to 50% (19, 22, 27). Interestingly, a metanalysis on 7616 patients highlighted a higher risk for MetS in schizoaffective disorder compared to schizophrenia (26). Conclusive evidence on the prevalence of MetS in other PSD is lacking.

The wide heterogeneity in prevalence reporting may depend on numerous factors, such as the aforementioned geographical, genetic, and gender differences, potential discrepancies in diagnostic criteria for MetS and specific PSD, and, importantly, on differences in comorbidity and medications, as discussed in the next section.

## 3 Pathogenesis and pathophysiology of metabolic syndrome in psychosis-spectrum disorders

The pathogenesis and pathophysiology of MetS in patients with PSD is complex and multifaceted, with multiple contributing factors. The relationship between MetS and PSD remains enigmatic, with different viewpoints surrounding whether MetS acts as a comorbidity, a consequence, or a confounding factor in the pathophysiology of PSD. While MetS pathogenesis and pathophysiology is beyond the scope of this review and will only be briefly reviewed, we will highlight in the following sections the pathophysiological aspects that are most relevant to PSD patients.

### 3.1 Antipsychotics increase the risk of metabolic syndrome

**Figure 1:**
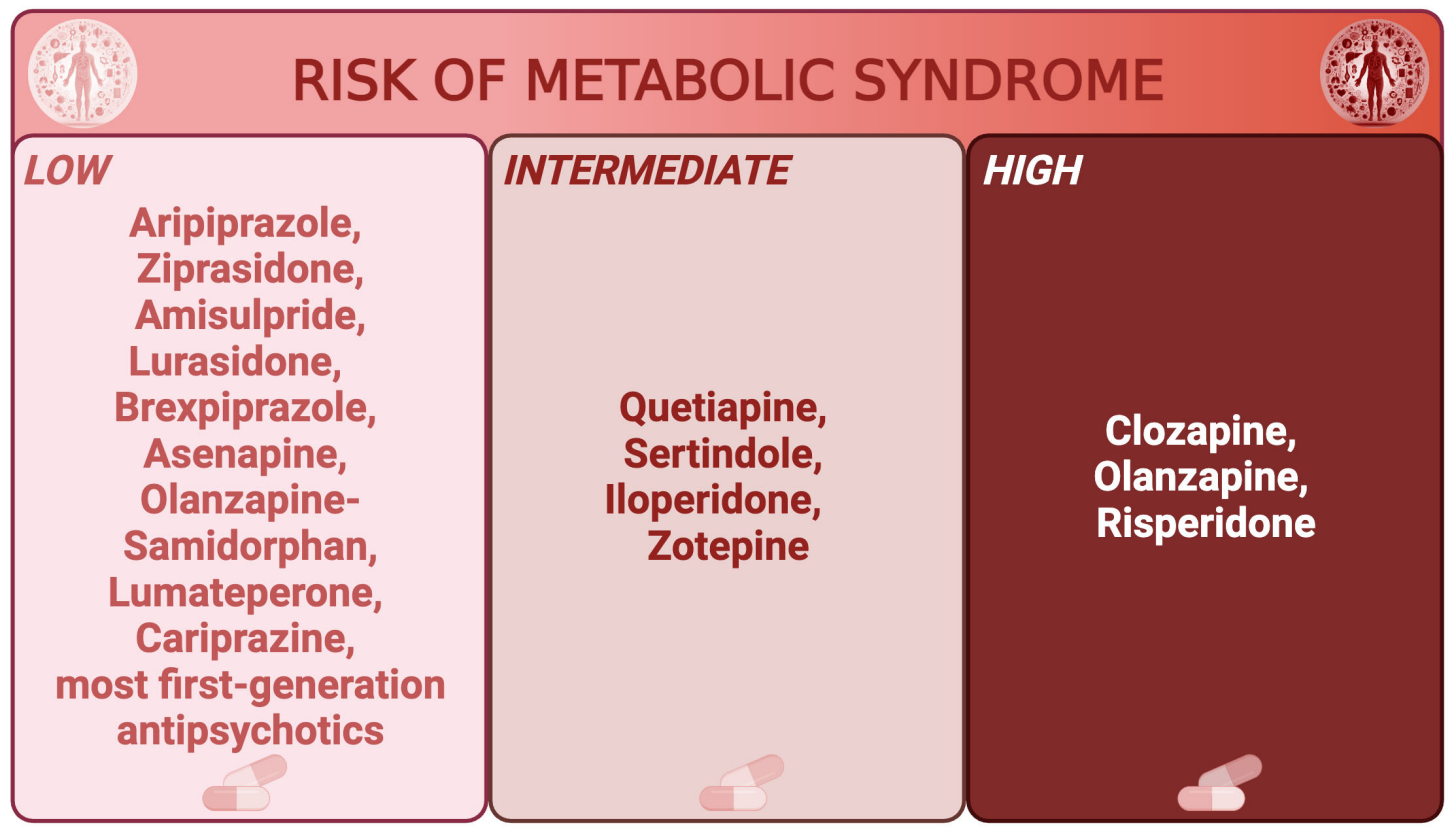
Main antipsychotics classified by risk of metabolic syndrome. Main antipsychotics are divided in low, intermediate, and high risk of metabolic syndrome.

Antipsychotic medications have gained attention for their increased risk of MetS. Indeed, the prevalence of antipsychotic-related MetS ranges from 23 to 50 percent depending on the sample, but prolonged use of almost all antipsychotics, and especially second-generation ones, is associated with MetS and weight gain to varying degrees (28–30), even in younger populations (31). Indeed, the rate of MetS was highest for clozapine (51.9%), olanzapine (28.2%) and risperidone (27.9%) in the aforementioned metanalysis on 25,692 schizophrenia patients (19). **Figure 1** classifies the main antipsychotics based on their risk of metabolic syndrome (32).

### 3.2 Metabolic syndrome may precede antipsychotic treatment in PSD patients

Evidence from antipsychotic-naïve PSD patients suggest a direct link between PSD and MetS that could be, at least partly, independent of antipsychotic usage. For instance, antipsychotic-naïve patients with schizophrenia and schizoaffective disorders exhibit higher insulin resistance compared with matched controls (33), and specific dysregulations of metabolic parameters, including high-density lipoprotein cholesterol and homocysteine, have been identified in treatment-naive FEP patients (34). Similarly, a meta-analysis of 23 studies found that waist-to-hip ratio was increased in antipsychotic-naive and minimally treated PSD patients compared to healthy controls (35).

Another interesting hypothesis involve the fact that early-life stress (ELS) and adversities have been found to increase the risk for developing PSD in a meta-analysis of prospective and cross-sectional case-control studies on almost eighty thousands subjects (36). This finding supported by significant gene-environment correlation between polygenetic risk scores for schizophrenia and childhood adversity, highlighted in another meta-analysis (37). Similarly, severe stress during adolescence has been suggested to contribute to risk of greater waist circumference in early psychosis (38). ELS is associated with a pro-inflammatory state in the general population (39) as well as in psychiatric and PSD patients (40, 41). In fact, not only is inflammation a pivotal component of MetS (42, 43), but it is also closely related with PSD pathophysiology (40, 44–46), even in FEP patients (47). Together, these findings suggest that PSD patients may present a specific vulnerability to MetS due to ELS and to the associated pro-inflammatory state, which both precede psychiatric diagnoses and antipsychotic treatments.

### 3.3 Metabolic syndrome and inflammation are interrelated

The interplay between chronic inflammation, MetS and obesity is established through various studies (42, 43). MetS is characterized by an elevated inflammatory state [heightened levels of inflammatory molecules such as C-reactive protein, tumor necrosis factor-alpha, and interleukins 6 and 18 (43)] independent of obesity (48). In fact, adiponectin, an anti-inflammatory adipokine, is decreased in MetS, and its decline parallels the increase in the number of MetS components an individual exhibits, coinciding with elevated inflammatory markers (48, 49). Visceral adipose tissue functions as both a paracrine and an endocrine organ, secreting proinflammatory and atherogenic adipokines such as leptin and tumor necrosis factor-alpha, along with anti-inflammatory adipokines like adiponectin (50). Dysregulation of adipokine secretion, coupled with infiltration of macrophages into adipose tissue, leads to chronic low-grade inflammation associated with insulin resistance and type 2 diabetes (51). Insulin resistance is intimately tied to hypertension through various, multifactorial mechanisms, involving endothelial dysfunction stemming from free fatty acid-induced generation of reactive oxygen species, as well as hyperinsulinemia-induced activation of the sympathetic nervous system and inhibition of nitric oxide synthase, alongside effects mediated by adipose tissue-derived cytokines (52, 53). Obesity is accompanied by hyperactivity of the renin-angiotensin-aldosterone system, further compounding hypertension (54). Indeed, excessive visceral fat is associated with heightened insulin resistance (53), a pivotal factor in MetS pathophysiology (1). The increased volume of expanded adipose tissue results in an augmented release of free fatty acids into the portal circulation, which are then transported to the liver and stored as triglycerides, substantiating the “portal theory” of MetS (53, 55). Insulin resistance disrupts the regulation of lipolysis, causing an elevated release of free fatty acids into the bloodstream (1). Hepatic insulin action is impaired due to the greater influx of free fatty acids, leading to elevated gluconeogenesis and subsequent hyperglycemia (56) (53). Peripheral insulin resistance in muscle tissue contributes to reduced glucose disposal (57). Over time, the pancreatic beta cells face increased demands to counteract resistance, ultimately resulting in increased risk of type 2 diabetes mellitus. As suggested in the previous section, these mechanisms are particularly relevant in the context of PSD, but consistent evidence supports the relationship between MetS, inflammation, and severe mental illnesses in general (58, 59).

### 3.4 Common and specific environmental factors modulate metabolic syndrome risk in PSD patients

In addition to these mechanisms, PSD patients exhibit distinct lifestyle patterns that contribute to the heightened risk of MetS within this population. More in detail, research has consistently shown that individuals with PSD tend to have dietary habits characterized by high consumption of processed foods, saturated fats, and refined sugars (60). These dietary choices not only contribute to obesity, a core component of MetS but also lead to dyslipidemia and insulin resistance. It is crucial for healthcare providers to assess and educate PSD patients about the importance of balanced nutrition, emphasizing whole foods, fruits, vegetables, and lean proteins, while reducing the intake of processed and sugary items.

Smoking, a prevalent risk factor for MetS, is of particular concern in PSD patients. Studies have shown that individuals with PSD have higher smoking rates than the general population. This not only contributes to an increased risk of MetS but also intensifies the cardiovascular risk inherent to both smoking and MetS. It is imperative for clinicians to prioritize smoking cessation interventions among PSD patients to mitigate these compounding risks. Notably, Mendelian randomization studies suggested a link between cigarette smoking and BD or schizophrenia (45), and individuals with these disorders tend to consume a greater number of cigarettes per day compared to the general population (61) and up to 75% of schizophrenic patients are smokers as opposed to 25% in the general population (62). It has been reported that individuals with schizophrenia who smoke engage in less physical exercise compared to nonsmokers, further exacerbating the risk of cardiovascular disease (61). Finally, tobacco users with PSD are more inclined to consume alcohol and caffeine on a daily basis, while also being less likely to avoid high salt and saturated fat intake (61).

Sedentary behaviors are another significant contributor to the development of MetS in PSD patients. Many individuals with PSD lead relatively inactive lives due to symptoms of their disorder, medication side effects, or lack of motivation. The consequences of sedentary lifestyles extend beyond weight gain and include decreased insulin sensitivity, muscle mass loss, and worsening of MetS components (60). Interventions that promote physical activity tailored to the needs and abilities of PSD patients are crucial. Collaborative efforts between mental health and physical health professionals can develop exercise programs that not only improve metabolic health but also positively impact the overall well-being and symptom management of individuals with PSD (60). Overall, sedentary behavior, unhealthy diet, and smoking thus contribute to MetS and cardiovascular risk among PSD (61–63). Recognizing and addressing these lifestyle factors are essential steps in reducing the prevalence and severity of MetS in this vulnerable population, as discussed in Sections 4.2. and 5.

## 4 Clinical implications

MetS in patients with PSD carries significant clinical implications, encompassing both shared and distinctive features compared to the general population.

In general, MetS carries a heightened cardiovascular and type 2 diabetes mellitus risk, and the combined presence of MetS components may exacerbate these risks beyond the individual risk factors (64–67). Other conditions associated with MetS are obstructive sleep apneas (68, 69), hypogonadism (70, 71), chronic kidney disease (72), cancers (such as colon, pancreas, kidney, prostate, endometrial, and breast cancer) (73), Nonalcoholic Fatty Liver Disease (NAFLD) (74), hyperuricemia and gout (75), and Polycystic Ovarian Syndrome (PCOS) (76). In particular, PCOS is associated to a higher prevalence of PSD, such as BD (77, 78) or schizophrenia (79). Additionally, multiple components of MetS, including hypertension, hyperlipidemia, and diabetes, associate with an elevated risk of cognitive decline and dementia, especially when accompanied by high inflammation levels (80).

Several factors complicate the management of these risk factors in PSD patients with MetS. These include, first of all, the well-documented stigma and lower quality of somatic care offered to psychiatric patients (81), particularly those with PSD (82).

Secondly, psychiatric symptoms, and especially psychotic ones, may hinder the long-term monitoring of somatic comorbidities and MetS. Even during non-acute phases, residual symptoms may disrupt proper follow-up, with schizophrenia and schizoaffective disorders potentially harboring residual psychotic symptoms (such as persecutory delusions involving healthcare professionals). Similar challenges are present for residual cognitive or depressive symptoms in BD, which could lead patients to miss medical appointments.

Finally, the environmental and idiosyncratic factors, outlined in the previous section, render PSD patients uniquely vulnerable to MetS risks, exemplified by their nearly twofold elevated risk of early cardiovascular-related mortality (83).

### 4.1 The deleterious impact of metabolic syndrome on psychiatric outcomes in specific PSD

MetS itself might exert clinical repercussions on psychiatric prognosis and symptoms in PSD patients. For instance, its influence on the course of BD results in more adverse outcomes, including reduced quality of life, heightened complexity of the disease, rapid cycling, increased suicide risk, functional impairment, and suboptimal treatment response (84). On the same line, obesity has been associated with various adverse outcomes in individuals with BD, including an elevated frequency of manic and depressive episodes over the course of their lives, more challenging-to-treat index mood episodes, a heightened recurrence rate of mood episodes, a greater tendency towards depressive episodes, and a shorter duration until relapse, as compared to their non-obese counterparts (85, 86). Consequently, physical comorbidity and MetS has been identified as a significant risk factor for psychiatric readmissions and other unfavorable outcomes in BD (87).

Similarly, a positive correlation has been established between body-mass index (BMI) and the severity of positive symptoms in drug-naïve FEP schizophrenia patients (88), and there is emerging evidence linking cognitive impairment in schizophrenia to metabolic dysfunction (89). Indeed, a recent meta-analysis highlighted that individuals with schizophrenia who also have MetS or diabetes mellitus tend to experience more severe cognitive deficits, and that there is a significant relationship between cognitive impairment in schizophrenia and the individual components of MetS (including hypertension, dyslipidemia, abdominal obesity and diabetes), suggesting that MetS may contribute to functional decline in these patients (90). Similarly, preliminary evidence suggests that also in individuals with BD MetS is associated with a higher prevalence of impaired executive function (encompassing critical aspects like action planning, inhibition, and impulse control) (91). Hence, interventions targeting obesity and cardio-metabolic risk could have dual benefits on both cardiovascular health and cognitive and functional disability related to PSD.

### 4.2 Managing metabolic syndrome in patients with psychosis spectrum disorders

From what has been discussed above, it is clear that managing MetS in patients with PSD presents unique challenges and considerations that differentiate their care from the general population. Despite this complexity, the following paragraph will summarize the main pillars to guide clinical care of this life-threatening syndrome.

First of all, to mitigate the risk of developing MetS, it is crucial to avoid, when possible, antipsychotic medications known to increase the risk of weight-gain and metabolic complications, such as clozapine and olanzapine (32), in patients experiencing a first psychotic episode. When clinical arguments support a role of antipsychotic medication in MetS development, adaptation of pharmacological treatment should be considered (28, 92, 93). Dose reduction of antipsychotics should be explored with caution, considering the patient’s history, clinical status, and potential for symptom exacerbation. Switching to antipsychotic drugs with lower weight gain and dyslipidemia risks, such as aripiprazole or ziprasidone, might promote weight loss and improve lipid profiles (28, 92, 93). Close clinical monitoring during antipsychotic switching is crucial. Gradual cross titration and a monitoring period of two to three months are recommended to assess efficacy. Interestingly, a recent dose-response metanalysis highlighted antipsychotic-specific metabolic signatures at specific dosages, which should be considered when adjusting antipsychotic dosages in PSD patients (94).

Lifestyle interventions customized for PSD patients can aid in weight loss and metabolic improvement (95). However, their efficacy remains variable (95). Structured interventions focusing on health education, physical activity, and active monitoring may yield better outcomes.

As in the general population, hyperglycemia/diabetes, dyslipidemia and blood pressure necessitate vigilant monitoring and patients with new-onset diabetes should be referred to primary care or an endocrinologist depending on the context (96).

Tobacco smoking cessation in PSD patients emphasizes varenicline and nicotine replacement therapy (97). Varenicline exhibits greater efficacy compared to nicotine replacement therapy or bupropion (97). Patient-specific factors should guide medication selection (98–104). For instance, antidepressants may improve negative symptoms in schizophrenia and schizoaffective disorder, but their use must be approached cautiously, especially considering the risk of iatrogenic mood episodes.

As a second-line intervention, pharmacological treatment of metabolic alterations and weight loss treatment in PSD patients may be considered and is discussed in detail elsewhere (96, 105–108) and in Section 4.4. Concerning non-pharmacological and mixed interventions for metabolic dysregulation in PSD, a systematic review of 23 randomized controlled trials suggested that cognitive/behavioral interventions and pharmacological adjuncts both showed modest but significant effects in preventing weight gain. In terms of weight loss treatments, cognitive/behavioral interventions were more effective than standard care. However, the findings were limited by the small number of studies, small sample sizes, short study durations, and variability in interventions (109).

Finally, it should be noted that, due to the aforementioned potential challenges with patient follow-up and referrals, some PSD patients receive clinical care solely in mental health settings (110). Hence, collaborative approaches involving healthcare providers with expertise in metabolic abnormalities, internal medicine, endocrinology, and care management professionals can optimize treatment (110). These professionals facilitate clinician collaboration and patient navigation within complex healthcare systems (110).

### 4.3 Challenges in metabolic and cardiovascular risk assessment for early-stage PSD patients

It is important to highlight that metabolic and cardiovascular risk should be assessed and monitored also in PSD patients who do not have MetS. This aligns with the findings from the aforementioned meta-analysis, which revealed comparable MetS prevalence between drug-naïve FEP patients and controls. However, the FEP group demonstrated markedly higher prevalence of MetS components and cardiovascular risk factors (21). This implies that MetS might not effectively predict early cardiovascular risk in young psychosis patients, and we are lacking instrument to assess the early metabolic and cardiovascular risk in this population (21). Indeed, other cardiovascular risk predictors, such as the Framingham score for coronary cardiovascular disease risk or the SCORE, lack applicability for young psychosis patients, being primarily tailored for individuals aged 45 and above, and would underestimate cardiovascular risk in youths (21). One alternative algorithm for predicting cardiovascular risk in schizophrenia is PRIMROSE, validated in chronic schizophrenia patients, but also unsuitable for young individuals in early psychosis stages (111, 112). Indeed, while notable metabolic changes (e.g. triglyceride concentration, waist circumference, and high-density lipoprotein-cholesterol concentration) may be present in FEP individuals, and worsen over the course of the first following year, no conclusive predictors of MetS or of antipsychotic-induced weight gain (113) have been identified in this population, yet (114), and polygenetic risk scores-based predictions showed preliminary early promise but remain to be validated (115). Thus, individualized, longitudinal, and comprehensive surveillance of clinical and biological markers of metabolic and cardiovascular risk is necessary in PSD patients, and in FEP ones, who may be a higher risk of weight-gain and may benefit the most from preventive interventions.

This comprehensive risk profile should consider medical factors like obesity, dyslipidemia, hypertension, hyperglycemia, and established diabetes, as well as behavioral factors such as poor diet and smoking (19). This risk profile can guide ongoing monitoring, treatment selection, and management. Among the components of MetS, increased waist size, or abdominal obesity, appears to be a strong predictor of MetS in PSD. Waist size is closely associated with factors like hyperinsulinemia, dyslipidemia, and impaired glucose tolerance. Some evidence supports using waist size or BMI as a simple screening tool for MetS in schizophrenia and schizoaffective disorder, although specificity was found to be lower than sensitivity (55% and 92% respectively) (116). Indeed, to ensure effective monitoring, guidelines recommend measuring waist circumference and BMI regularly (at least quarterly or annually on the long term, depending on the guidelines) as minimum monitoring, especially for patients receiving atypical antipsychotics (19). While measuring waist circumference and BMI may seem straightforward, affordable, and relatively simple, it’s concerning that approximately 50% of patients in routine care lack recorded BMI measurements (76), and roughly 60% of inpatients may not undergo a comprehensive physical examination (19, 117).

### 4.4 Cardiovascular risk in MetS and management in PSD patients

Patients with MetS are known to have a higher risk of cardiovascular disease (CVD) (118–120). A large systematic review and meta-analysis on about 950,000 patients established that MetS is associated with a 2-fold increase in fatal and non-fatal CV outcomes, and a 1.5-fold increase in all-cause mortality (120). It is currently unknown whether the risk associated with MetS exceeds the risk associated with the sum of its individual components (121), but the higher the number of individual components of MetS, the greater the risk of CVD (122). MetS patients not only are at higher risk of developing myocardial infarction (MI) (122), but they also tend to have larger infarct size and more in-hospital complications (123). Of all the different components of MetS, hyperglicemia and BMI ≥28.0 kg/m^2^ are associated with major adverse cardiovascular events (MACE) in patients with MI aged <45 year (124). Moreover, MetS in acute coronary syndrome (ACS) is significantly more present in women (55.9%-66.3%) than in men (40.2%-47.3%) (125, 126).

PSD patients have a reduced life expectancy of 15–25 years as compared to the general population, mostly due to increased CV deaths (127). In a recent multicentric French study, 4,424 patients with schizophrenia were shown to develop CVD requiring hospitalization, such as MI, heart failure (HF) and stroke, at an early age, around ten years earlier than the general population (128). Importantly, these CV events are associated with a high percentage of preventable and treatable risk factors, such as hypertension (11.3%), obesity (9.7%), and diabetes (7.8%) (128). CVD in PSD patients may be caused by comorbidities, the adoption of unhealthy lifestyles in patients with inadequately treated PSD, and the use of antipsychotic drugs (129, 130).

Appropriate CV risk assessment and management is therefore crucial in patients with PSD. CV risk assessment may be estimated through different methods that generally show strong disagreement and may significantly underpredict CVD in PSD patients (131). However, based on current evidence, the assessment of MetS should be preferred as CV risk-estimator in mental healthcare as it is relatively easy and fast to use, and it is not restricted to older individuals (131). In particular, patients are considered to have MetS if they fulfil three or more of the following criteria (132): 1) waist circumference ≥ 88/102 cm (female/male); 2) systolic blood pressure ≥130 mmHg, or diastolic blood pressure ≥ 85 mmHg, or antihypertensive pharmacological treatment; 3) HDL-cholesterol <1.30/1.03 mmol/L (female/male) or receiving lipid-lowering drugs; 4) triglycerides ≥1.7 mmol/L or receiving lipid-lowering drugs; and 5) fasting glucose ≥6.1 mmol/L or receiving antidiabetic medications.

**Figure 2:**
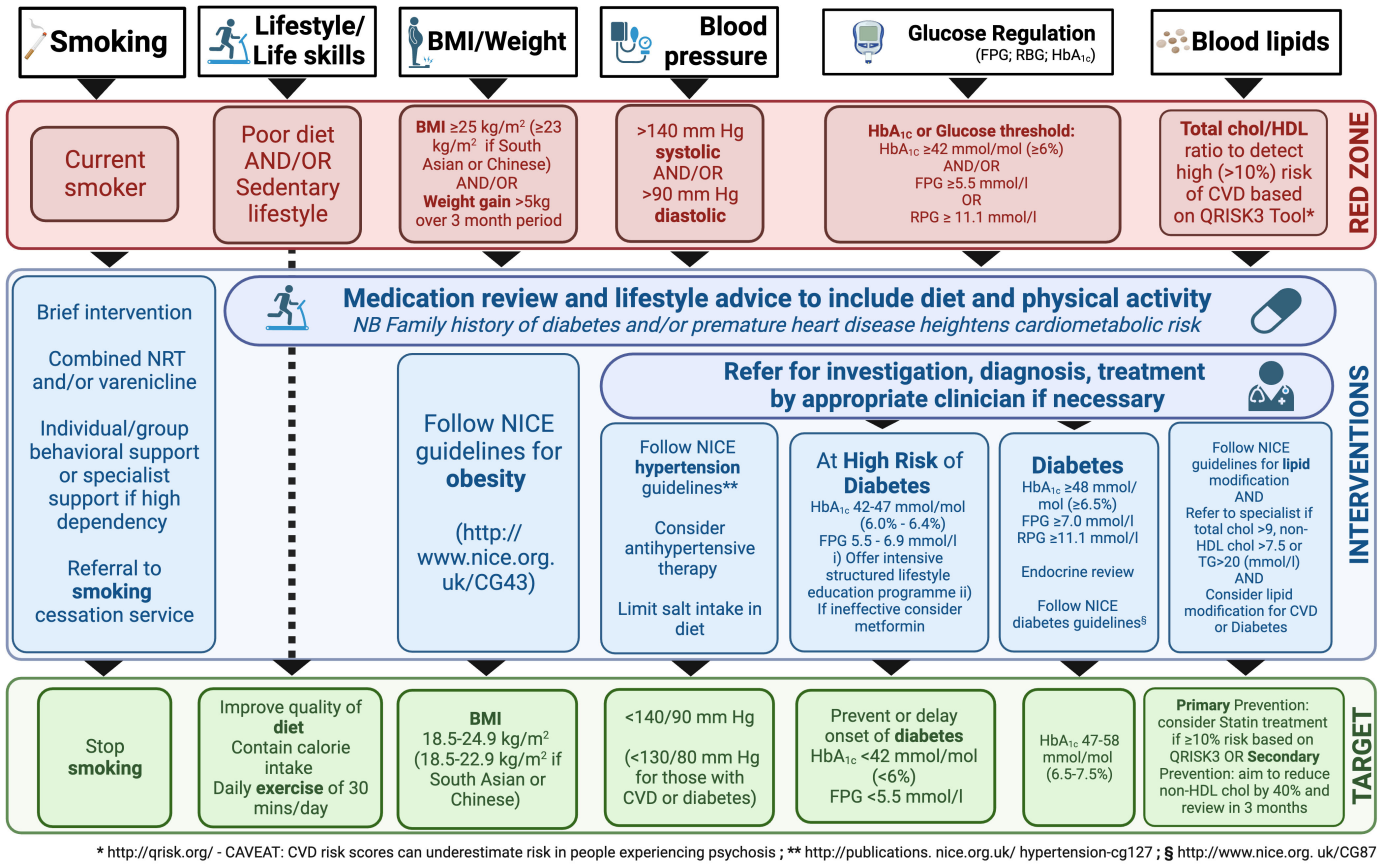
Resource for Promoting Cardiometabolic Wellness: A Framework for Interventions in Patients Taking Antipsychotic Medication. BMI, body mass index; chol, cholesterol; CVD, cardiovascular disease; FPG, fasting plasma glucose; HDL, high-density lipoprotein; NICE, National Institute for Health and Care Excellence; NRT, nicotine replacement therapy; RPG, random plasma glucose; TG, triglycerides. The figure is inspired from the Lester Cardiometabolic Health Resource guidelines (133).

Shared guidelines for CVD risk management in PSD patients should be introduced in the daily clinical practice of in- and out-patient psychiatric services, such as the Lester Cardiometabolic Health Resource (133), summarized in **Figure 2**.

At baseline, the specialist should look for a history of substantial weight gain (e.g., 5 kg), especially if rapid; assess smoking activity, exercise, and diet; and characterize family history (diabetes, obesity, CVD in first degree). At examination, weight, BMI, blood pressure (BP), and pulse should be recorded, whereas laboratory tests should include fasting estimates of plasma glucose (FPG), HbA1c, and lipid profile (total cholesterol, non-HDL, HDL, triglycerides).

As mentioned, negative symptoms (apathy, avolition, social withdrawal) need to be targeted and smoking cessation, a heart-healthy diet, physical activity (at least 30 minutes per day), and regular sleep routines need to be actively promoted (127, 134). Smoking, in particular, not only increases CV risk, but also complicates treatment, since the hydrocarbons in cigarette smoke accelerate the metabolism of dibenzodiazepines, clozapine and olanzapine (135). Weight should be assessed weekly in the first 6 weeks of taking a new antipsychotic, as rapid early weight gain may predict severe weight gain in the longer term.

If the patient has not successfully reached their targets after 3 months, pharmacological interventions such as anti-hypertensive, lipid-lowering, and diabetes therapy should be considered, according to current recommendations (96, 105–108). For instance, potential approaches may include metformin, topiramate, aripiprazole augmentation for clozapine patients, or liraglutide, if necessary (96, 105–108). Metformin is often preferred for its tolerability and effectiveness (105). Indeed, a systematic review of 17 randomized controlled trials involving 1,388 participants, various medications, including metformin, H2 antagonists, and monoamine modulators, showed potential for preventing weight gain and BMI increase in people with limited certainty of evidence, while topiramate did not appear effective; however, further research is needed to confirm these findings (89). As always, careful consideration of efficacy, side effects, dosing, and contraindications is required when choosing medication. Notably, some antipsychotics (i.e. clozapine, loxapine, haloperidol, melperone, risperidone, and olanzapine) are known to cause significant creatine kinase (CK) elevations (136). Therefore, it is prudent to check CK levels in PSD patients before adding a statin to antipsychotic treatment.

## 5 Conclusions, strengths, limitations, and perspectives

This narrative review provides a comprehensive overview of the current state of knowledge regarding MetS in individuals with PSD (**Table 1**). Fundamental concepts surrounding MetS in PSD revolve around the importance of early identification and management to mitigate the long-term cardiovascular and metabolic consequences. While there is consensus on the need for early intervention, questions persist about the most effective strategies for achieving this goal. BMI and waist circumference emerge as reasonable and simple screening measures, but their specificity may be low and more personalized and extensive monitoring may be needed depending on the populations (19). Depending on the clinical context, assessments should also include weight history, smoking, exercise, diet, family history, blood pressure, pulse, and laboratory tests (at least FPG, HbA1c, and lipid profile). The role of healthcare professionals in multidisciplinary teams, including psychiatrists, physicians, nurses, and other specialists, is vital in educating and motivating PSD patients to make lifestyle improvements. Nonetheless, the challenges of implementing and sustaining the aforementioned behavioral interventions, such as smoking cessation, dietary enhancements, and exercise regimens, must be considered. While the aforementioned Lester Cardiometabolic Health Resource (133) provides useful guidelines for managing cardiovascular and metabolic risk in PSD patients, there are ongoing debates regarding the monitoring instruments for MetS risk and the ideal balance between lifestyle modifications and pharmacological interventions in managing MetS in PSD.

**Table 1:** Main evidence on MetS in PSD.

Disorder	MetS prevalence	Main putative risk factors for MetS	Factors associated with MetS
**PSD**	∼30%	Sedentary lifestyle Antipsychotic medications Genetic predisposition Substance abuse and addiction (e.g. smoking) Diet Pro-inflammatory state Stress Insufficient somatic care	**↑**morbidity **↑** mortality **↑**cardiovascular risk
**SCZ**	∼50%		**↑** cognitive impairment **↑** positive symptoms
**SCZaff**	∼70%		
**BD**	∼60%		**↑** rapid cycling **↑** suicide risk **↓**executive function

Prevalence, risk factors, and implications of MetS in PSD. BD, Bipolar Disorder; MetS, Metabolic Syndrome; PSD, Psychotic Spectrum Disorder; SCZ, Schizophrenia; SCZaff, Schizoaffective disorder. References are provided in the text.

Upward arrows indicate an increase, downward arrows a decrease.

The transdiagnostic approach of this review opens avenues for future research MetS in PSD, which have relevant clinical implications for monitoring and interventions. For instance, by recognizing common vulnerabilities that transcend diagnostic boundaries, a transdiagnostic perspective allows for the identification of shared risk factors and early indicators in youths at high risk for PSD, that could evolve towards distinct PSD (e.g. BD or schizoaffective disorder). Hence, by addressing shared risk factors transdiagnostically, clinicians and researchers can pave the way for more effective and personalized early interventions, ultimately shaping a more nuanced and proactive approach to the diverse trajectories that high-risk youth may traverse within the spectrum of PSD. In this sense, a transdiagnostic approach may potentially guide tailored early or preventive interventions targeting metabolic dysregulation across different PSD, aiming at mitigating the long-term metabolic and psychiatric outcomes. Indeed, this review highlights the importance of early identification and management of MetS in PSD patients, emphasizing the need for a multidisciplinary approach and providing a holistic view of the topic. Inclusion of recent research findings ensures that the information is up-to-date and relevant. However, limitations must be acknowledged. Firstly, as a narrative review, the article lacks the systematic rigor of a meta-analysis or systematic review, and no quantitative synthesis was performed. Furthermore, the examined literature on MetS in PSD presents itself some limitations. These include the lack of conclusive studies on MetS in various PSD subtypes, such as delusional disorder, schizotypal personality disorder, schizophreniform disorder, brief psychotic disorder, and psychosis associated with substance use or other medical conditions. While some evidence exists on schizoaffective disorder, it is less studied than schizophrenia and bipolar disorder. This lack of research hinders our understanding of how MetS manifests and progresses in different PSD populations, impeding the development of tailored interventions for these specific groups. Additionally, some studies employ cross-sectional designs, hindering the establishment of causation and the determination of directional relationships. Variability in diagnostic criteria for both MetS and PSD further complicates comparisons between studies. Confounding factors such as medication use, lifestyle, and socioeconomic status may not always be adequately addressed. Publication bias can distort the overall understanding of this relationship. The biological mechanisms linking MetS and PSD remain incompletely understood, and more research is needed in this area. Future research should address these limitations to advance our understanding in this field. Similarly, further research into a suitable scoring system for assessing MetS risk in young FEP patients is desperately needed, as this population may benefit the most from early interventions and seems particularly vulnerable to MetS.

In light of these challenges and gaps, potential developments in the field of MetS in PSD emerge. Researchers and healthcare professionals should collaborate to explore novel, PSD-specific interventions and community resources that can effectively address MetS and its associated risks (96). Ongoing research into non-pharmacological interventions for MetS should be encouraged and expanded (137). The present transdiagnostic approach aims at paving the way to further research investigating for instance the role of shared biological or psychosocial factors, such as stress and lifestyle behaviors, in the development of MetS across PSD, and to randomized controlled trials to assess the efficacy of transdiagnostic interventions, such as lifestyle modifications and psychosocial therapies, for reducing MetS risk and improving overall well-being in individuals with PSD.

Indeed, the recognition of commonalities among PSD underscores the importance of shared interventions to address factors that cut across specific disorders. However, it’s equally crucial to emphasize the development of personalized treatment approaches that take into account individual and disorder-specific differences based on genetic, environmental, and pharmacological factors. The future of research in this field should aim to enhance and add specificities to transdiagnostic interventions. By tailoring treatments to the unique characteristics and needs of each patient, we can potentially improve treatment outcomes and the overall well-being of individuals with PSD.

## Author contributions

LS: Conceptualization, Investigation, Methodology, Writing – original draft, Writing – review & editing, Data curation, Formal analysis, Software, Visualization. AA: Supervision, Writing – original draft, Writing – review & editing. GP: Writing – original draft. OS: Conceptualization, Methodology, Supervision, Writing – review & editing.
